# Quantitative Assessment of Soluble Carbohydrates in Two Panels of Pulses (*Phaseolus vulgaris* and *Pisum sativum*) Using Ultrasound-Assisted Extraction (UAE) and HPLC

**DOI:** 10.3390/foods15020391

**Published:** 2026-01-21

**Authors:** Roberto Rodríguez Madrera, Ana Campa Negrillo, Juan José Ferreira Fernández

**Affiliations:** 1 Área de Tecnología de los Alimentos, Regional Agrifood Research and Development Service (SERIDA), E-33300 Villaviciosa, Asturias, Spain; 2 Área de Cultivos Hortofrutícolas y Forestales, Regional Agrifood Research and Development Service (SERIDA), E-33300 Villaviciosa, Asturias, Spain; anamaria.campanegrillo@asturias.org (A.C.N.); juanjose.ferreirafernandez@asturias.org (J.J.F.F.)

**Keywords:** pulses, legumes, soluble carbohydrates, RFOs, UAE, nutritional profiling, breeding programs, sustainable analytical methods

## Abstract

Pulses (edible dry seeds from legumes) are among the most important crops worldwide. These legumes contain a diverse range of carbohydrates, some of which, such as RFOs (raffinose family oligosaccharides), are considered antinutritional factors due to their negative impact on digestion. An analytical method based on high-power ultrasound-assisted extraction and HPLC analysis was developed and validated for the quantitative determination of soluble carbohydrates (verbascose, stachyose, raffinose, sucrose, galactinol, glucose, galactose, fructose, and myo-inositol) in common beans (*Phaseolus vulgaris*) and peas (*Pisum sativum*). The proposed method is fast (extraction time: 1 min), reproducible (RDS: 6.9%), accurate (97.5%), and environmentally sustainable. The method was applied to local collections of *P. vulgaris* (*n* = 12) and *P. sativum* (*n* = 34), revealing similar qualitative profiles but notable quantitative differences. In *P. vulgaris*, sucrose and stachyose were predominant, while in *P. sativum*, verbascose stood out. The total sugar content was higher in peas, especially in commercial varieties, which also showed elevated sucrose levels. Some local varieties combined high sugar content with favorable relative levels between RFOs and other sugars, making them valuable candidates for breeding programs. Linear discriminant analysis enabled classification and prediction of species and varieties, confirming the usefulness of soluble carbohydrates as tools for characterizing these plant materials.

## 1. Introduction

Pulses are an important source of plant-based nutrients due to their high content of protein, carbohydrates, vitamins, and minerals, and their low fat content. They also provide notable levels of functional components, such as dietary fiber and phenolic compounds [1,2,3,4]. As a result, among other health benefits, the consumption of pulses is suitable for individuals with diabetes. Moreover, it may contribute to reducing the risk of coronary heart disease and neural tube defects, such as spina bifida, help prevent iron deficiency, and improve the protein quality of vegetarian diets [5].

The carbohydrate content of pulses is predominantly composed of slowly digestible starches with a low glycemic index, dietary fiber, and variable amounts of soluble carbohydrates, which depend on the species [6,7]. These soluble carbohydrates are mainly composed of raffinose family oligosaccharides (RFOs), which are characterized by the presence of one or more galactose molecules, sucrose, and small amounts of monosaccharides. Humans are unable to metabolize galactose, which prevents its degradation in the stomach and small intestine. As a result, the gut microbiota in the large intestine converts it into its corresponding monomers and subsequently carries out anaerobic fermentation, producing various metabolites, including gases such as CO_2_, H_2_, and methane, which are responsible for flatulence. For this reason, the presence of RFOs has traditionally been considered as an antinutritional factor. However, recent studies have increasingly emphasized the beneficial role of these sugars as fermentable substrates for the growth and activity of the intestinal microbiota [8]. Other soluble carbohydrates, reported in variable levels depending on the species, include cyclitols (cyclic polyols), among which myo-inositol and various galactosyl cyclitols are particularly noteworthy. In this sense, it is important to highlight that RFOs and cyclitols play a key role in cellular metabolism, as they act as reserve molecules and cellular protectants against abiotic stress such as cold and drought. They also contribute to membrane stability during desiccation and germination, thereby influencing seed vigor. Consequently, these metabolites have been incorporated into breeding programs [9].

The determination of soluble carbohydrates in pulses has been addressed by various authors using different chromatographic techniques; previously, aqueous extraction has been used. Due to their high solubility in water, this solvent has generally been used, often in combination with organic solvents, such as ethanol, to prevent the co-extraction of polysaccharides and proteins that may interfere with chromatographic analysis. The extraction of soluble carbohydrates is typically conducted at varying temperatures over prolonged periods, and in certain cases, multiple extraction cycles are necessary [10,11,12,13]. However, it is well established that extraction at temperatures between 30 and 45 °C promotes the hydrolysis of seed α-galactosides [14], primarily releasing galactose. This suggests that extraction should be performed at temperatures above 60–65 °C to inhibit seed enzymatic activity [15,16]. Moreover, this hydrolysis becomes more pronounced as extraction time increases, indicating the need to minimize extraction duration. In this context, the use of high-power ultrasound (frequency > 20 kHz), equipped with an immersion probe that directly transmits ultrasonic energy to the extraction medium, represents an effective alternative to conventional methods such as stirring or ultrasonic bath extraction. This technique enables shorter extraction times and lower energy consumption while achieving quantitative and reproducible extractions [4,17,18].

Subsequently, the extracts are analyzed by high-performance liquid chromatography (HPLC), using different types of columns (reverse phase and ionic exchange), mobile phases (water and organic solvents), and detectors (refraction index, photodiode array, evaporative light scattering, and mass spectrometry). Gas chromatography (GC) is less commonly employed, as it requires prior derivatization of the sugars [2,10,12,13,19,20,21]. In this regard, it is worth noting that although analytical methods employing different detection systems have been proposed, considering the reported concentration levels of these analytes, the use of a universal detector such as the refractive index (RI) appears to be an appropriate choice. Furthermore, RI detectors are widely used in analytical laboratories due to their robustness, ease of maintenance and operation, straightforward interpretation of results, and cost-effectiveness, making them particularly suitable for characterization studies involving large sample sets.

In general, methods used to determine soluble carbohydrates in pulses focus exclusively on the major sugars (verbascose, stachyose, raffinose, and sucrose) and are often applied under conditions optimized for other matrices without prior evaluation of their suitability. Therefore, we consider it essential to employ an optimized and validated method—accurate, reproducible, and with well-defined detection limits—for the analysis of soluble carbohydrates, including the monosaccharides, disaccharides, oligosaccharides, and cyclitols present in the pulses of interest. Furthermore, the use of a validated method would enable the development of a robust database with applications in various fields, such as the formulation of new products based on dry seeds, the genetic improvement of varieties, or the selection of varieties with greater nutritional value.

Common bean (*Phaseolus vulgaris*) and pea (*Pisum sativum*) are two legume species cultivated worldwide, together covering an area of 4.78 Mha in 2024 [22] and encompassing a wide range of varieties. In Asturias (northern Spain), there is a large tradition of cultivating these legume species, which are deeply embedded in the region’s economy and cultural heritage. This has resulted in a natural selection of species well adapted to the environment, making them especially valuable in the current context of seeking sustainable food systems.

This study aimed to develop and validate an analytical method for the quantification of soluble carbohydrates (monosaccharides, disaccharides, oligosaccharides, and cyclitols) in legumes and to apply it for the characterization of two local seed collections of common bean and pea, thereby providing baseline data to support future breeding programs. 

## 2. Materials and Methods

### 2.1. Sample Material and Preparation of the Extracts

A total of 34 pea (*P. sativum*) accessions (21 local and 13 commercial) and 12 local common bean (*P. vulgaris* L.) accessions, maintained in the germplasm collection of the Regional Service for Agri-Food Research and Development (SERIDA), were analyzed. The plants were cultivated in the field and the seeds, dried on the plants in open air, were stored under vacuum, protected from light, at −20 °C until analysis. Pulses (50 g per accession) were milled at the moment of extraction in a coffee grinder and the powders were sieved through a standard sieve (number 18, corresponding to a sieve open ring size of 1.00 mm).

Samples (0.25 g of flour) were extracted using 50 mL of boiling water for 1 min in 100 mL capacity vials and were placed in a water bath at 70 °C to inhibit enzymatic hydrolysis [15]. A 7 mm sonotrode was used to perform the extraction at 100% amplitude (37 W power output). After extraction, the solids were separated from the mixture by centrifugation for 10 min at 5000× *g*. The supernatant was dried using a rotary vacuum evaporator at 50 °C; the residue was redissolved in 5 mL of water and then filtered through a prewashed C18 cartridge and a 0.45 μm cellulose acetate membrane. Analytical determinations, including extraction and chromatographic analysis, were performed in duplicate.

### 2.2. Reagents and Standards

Verbascose, stachyose, raffinose, sucrose, glucose, galactose, fructose, mannitol, galactitol, sorbitol, and myo-inositol were supplied by Sigma-Aldrich (St. Louis, MO, USA). Galactinol was purchased from Fluorochem (Hadfield, UK). Ethanol was purchased from Panreac (Barcelona, Spain) and was of analytical grade. Water was purified using a Milli-Q system from Millipore (Bedford, MA, USA).

### 2.3. HPLC Analysis

The determination of soluble carbohydrates—verbascose, stachyose, raffinose, sucrose, galactinol, glucose, galactose, fructose, and myo-inositol—in the extracts was performed using an HPLC system (Waters Associates, Milford, MA, USA) equipped with a 510 pump, a 717 Plus autosampler, a temperature controller, and a 410 refractive index (RI) detector. Separations were carried out on a cation-exchange column (Sugar-Pak I, Waters Associates) using an aqueous solution containing 50 mg/L of the calcium salt of EDTA as the mobile phase at a flow rate of 0.3 mL/min. The injection volume was 10 µL and the column oven temperature was set to 90 °C.

MS spectra were recorded using a Dionex Ultimate 3000 RS U-HPLC system (Thermo Fisher Scientific, Waltham, MA, USA) coupled to a micrOTOF-QII High-Resolution Time-of-Flight (UHR-TOF) mass spectrometer with Qq-TOF geometry (Bruker Daltonics, Bremen, Germany) and an electrospray ionization (ESI) source, under the separation conditions described above. The mass spectrometer operated in positive ionization mode, and spectra were acquired by scanning the mass range from *m*/*z* 50 to 2000. Nitrogen was used as the drying, nebulizing, and collision gas. The drying gas flow was set at 8 L/min at 200 °C, the nebulizer pressure at 1.2 bar, and the capillary voltage at 4500 V. Mass spectra were acquired in full scan (FS) and broad-band collision-induced dissociation (bbCID) modes. Collision energy for MS/MS was set at 40 eV.

The identity of the analytes was confirmed by comparing and combining their retention times and mass spectra, and was further validated using authentic standards when available. Quantification was performed by HPLC–RI according to an external standard method.

The instrumental limits of detection and quantification (expressed in mg/L) were estimated as 3 × Sa/m and 10 × Sa/m, respectively, based on the residuals of the calibration curves at low concentrations, where Sa is the standard deviation of the intercept values and m is the slope of the calibration curve y = a + mx. Method limits of detection and quantification (expressed in mg/g) were calculated from the instrumental values, taking into account the final extract volume (5 mL of water) and the mass of bean flour used for extraction (0.25 g).

### 2.4. Experimental Design and Validation Procedure

Ultrasonic-assisted extraction (UAE) was employed to extract soluble carbohydrates using an ultrasonic homogenizer UP200Ht (Hielscher, Teltow, Germany) equipped with a 7 mm diameter sonotrode, operating at a frequency of 26 kHz and a power input of 200 W. Extractions were carried out at 100% amplitude, corresponding to a power output of 37 W.

According to the analyte levels reported in the literature [9], a minimum sample mass of 0.25 g and a final volume of 5 mL were established to achieve the necessary sensitivity in the chromatographic analysis. Based on this, the optimization of extraction conditions was carried out following a factorial design with two factors: extraction time (three levels: 1, 2, and 3 min) and sample mass (two levels: 0.25 and 0.50 g). The rest of the conditions are described in Section 2.1.

The parameters evaluated for method validation were selectivity—assessed through MS spectra and analyte peak quality criteria—and precision, calculated as relative standard deviation (RSD%) for both repeatability and reproducibility. The accuracy of the method was evaluated in three ways: (i) exhaustive extraction, consisting of three consecutive extractions of the plant material; (ii) comparison of the results obtained with other extraction conditions reported in the literature; and (iii) analysis of legume samples obtained from a proficiency testing provider (ASFAC-LAB, Associació Qualimac, Barcelona, Spain).

### 2.5. Data Treatment

To detect significant differences between the levels of the factors evaluated during the optimization of extraction conditions, a two-way ANOVA was applied, considering extraction time and sample mass as factors. To assess significant differences among the plant materials analyzed, a one-way ANOVA was used. Pearson’s correlation coefficient was calculated to estimate correlations between variables. Linear discriminant analysis (LDA) was performed to identify the most discriminant variables between and within species, and for classification purposes. The programs used were SPSS version 15.0 (SPSS Inc., Chicago, IL, USA) and R software version 4.3.1 [23] for the results of the LDA through a discriminant function plot and a biplot.

## 3. Results and Discussion

### 3.1. Method Validation

#### 3.1.1. Chromatographic Separation and Identification

The predominant soluble carbohydrates in pulses are verbascose, stachyose, raffinose, and sucrose, with galactose, glucose, and fructose present at significantly lower levels, with marked differences observed between species for some of these compounds. For this reason, a SugarPak I cation-exchange column was selected due to its suitability for sugar separation using water as the mobile phase, in contrast to other options that require organic solvents [24,25].

**Figure 1:**
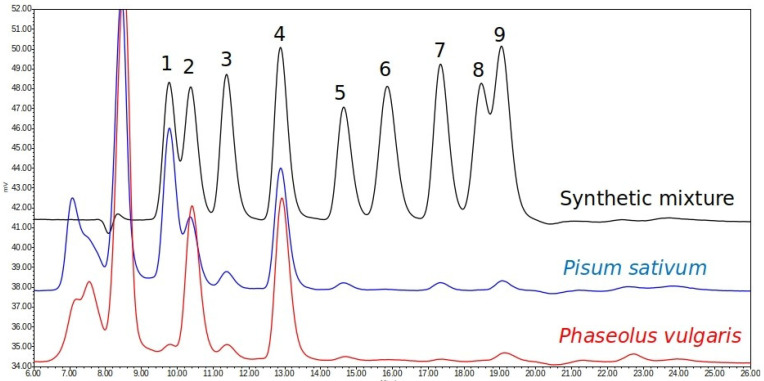
Typical chromatogram of soluble carbohydrates in *Phaseolus vulgaris*, *Pisum sativum* and a synthetic mixture. 1: verbascose, 2: stachyose, 3: raffinose, 4: sucrose, 5: galactinol, 6: glucose, 7: galactose, 8: fructose, and 9: myo-inositol.

As shown in Figure 1, under the chromatographic conditions used for routine analysis (10 μL of sample, isocratic flow at 0.3 mL/min, oven temperature of 90 °C, and RI detector), the seven soluble sugars were successfully separated and quantified in both species of interest, *P. vulgaris* and *P. sativum*. In all cases, mass spectrometry did not detect any contamination in these peaks, indicating that the selectivity of the analyses was adequate.

Moreover, mass spectrometry allowed the detection of a minor oligosaccharide in *P. sativum*, eluting before the verbascose peak, with an *m*/*z* of 1013.3167, corresponding to the sodium adduct of a compound with the molecular formula C_36_H_62_NaO_31_. This was accompanied by neutral losses of 162 Da, consistent with hexose units (Table 1). Considering its molecular formula, fragmentation pattern, and retention time, this compound could be ajugose (α-D-Galp-(1 → 6)-α-D-Galp-(1 → 6)-α-D-Galp-(1 → 6)-α-D-Galp-(1 → 6)-α-D-Glup(1 ↔ 2)-β-D-Fruf), a rarely reported oligosaccharide due to its low abundance in legumes, and a member of the RFOs [26]. Likewise, several polyols and their galactosides were successfully separated and identified, among which myo-inositol and galactinol (galacto-myo-inositol) were quantitatively predominant, both previously described in legumes. Other minor polyols, such as mannitol, galactitol, and sorbitol, were successfully separated and detected by mass spectrometry. However, their trace-level concentrations do not allow for analysis using a refractive index detector. Another cyclitol was tentatively identified in some samples of both *P. vulgaris* and *P. sativum*, with an *m*/*z* of 527.1591, corresponding to the sodium adduct with the molecular formula C_18_H_32_NaO_16_, accompanied by two neutral losses of 162 Da, consistent with hexoses. Considering its molecular formula, fragmentation pattern, and retention time, this compound could be an isomer of digalacto-myo-inositol, previously reported in legumes [27].

#### 3.1.2. Linearity and Limits of Detection and Quantification

The calibration curves showed good linearity (R^2^ > 0.999) for all analytes within the concentration ranges of the analyzed samples. The instrumental limits of detection and quantification, calculated from the curves at low concentrations, ranged between 2 and 8 mg/L and 7 and 26 mg/L, respectively (Table 2), in accordance with the values reported for HPLC-RI, HPLC-UV, and HPLC-ELSD systems [28,29]. Although other authors have reported significantly lower LOD and LOQ values using more sensitive detectors, such as MSD and PAD [12,13,30], the validated method using an IR detector allows for satisfactory quantification of the sugars described in these pulses at concentrations above 0.15 mg/g (0.015%) for monosaccharides and above 0.5 mg/g (0.05%) for the remaining sugars (Table 2), which is consistent with values reported in the literature [7,9], and is achieved while benefiting from advantages of the RI detector, such as its robustness, lower cost, and minimal maintenance requirements. In any case, it should be noted that although more sensitive instrumental methods have been proposed for the analysis of extracts, studies on soluble carbohydrates in pulses rarely report contents above 0.1 mg/g (0.01%), which, in our opinion, would not justify the use of high-cost equipment except for during the analytical method validation process.

#### 3.1.3. Optimization of Extraction Conditions and Method Validation

Water was initially selected as the extraction solvent, as it is the most suitable for these analytes and the only one that is fully environmentally friendly. Moreover, it allows for a higher solvent-to-sample ratio, which facilitates quantitative extraction in a single step. Although satisfactory extractions have been reported using hydroalcoholic mixtures, these tend to promote the precipitation of polysaccharides and proteins, which hinder both the extraction process and subsequent chromatographic analysis, in such cases, multiple extractions are required due to the low solubility of sugars in ethanol [10,11,31]. In order to facilitate the solubility of sugars in the samples and inhibit potential enzymatic degradation [32], boiling water (50 mL) was used, and the extractions were carried out in a water bath at 70 °C. Moreover, taking into account the limitations imposed by the LOQ achievable with the RI detector, and in order to quantify the amounts reported in legumes, which are typically above 0.1 mg/g, a minimum sample amount of 0.25 g was established.

Based on these premises, the extraction was optimized using a sample of *P. vulgaris* (variety Cornell49242) through a two-way factorial analysis: extraction time, with three levels (1, 2, and 3 min) and sample amount, with two levels (0.25 and 0.50 g). The results did not show significant differences for the time factor (Table S1), whereas sample amount was significant for the extraction of verbascose and myo-inositol, with both analytes showing higher extracted quantities using 0.25 g of sample, which appears to be justified by a higher solvent-to-sample ratio. Therefore, an optimal extraction time of 1 min and a sample amount of 0.25 g were selected. Additionally, it should be noted that when reconstituting the dried extract with 5 mL of water, subsequent sample conditioning through C18 cartridges and filtration becomes more difficult when using 0.5 g of sample due to the higher amount of extracted polysaccharides and proteins.

Therefore, the optimal extraction conditions were established as follows: 0.25 g of sample, 50 mL of boiling water, extraction for 1 min in a water bath at 70 °C, centrifugation, evaporation to dryness, and reconstitution to a final volume of 5 mL. Under these conditions, the precision and accuracy of the method were evaluated.

The accuracy of the method, evaluated through exhaustive extraction, was assessed using common bean (*P. vulgaris*) and pea (*P. sativum*), due to the particular interest of these species for our research group. As shown in Figures S1 and S2, the first extraction was quantitative and similar, in terms of recovery, for the major components of both matrices, with values ranging from 95% to 99% for sugars and from 91% to 100% for the two cyclitols.

The suitability of the method was also evaluated by comparing the results obtained for different matrices (dry bean, pea, chickpea, and lentil) with the extraction conditions suggested by other authors (Table 3). On the one hand, the extraction method proposed by Kotha et al. [13], originally designed for sugar extraction from various matrices (dry beans, lentils, and peas) using water and an ultrasonic bath for 60 min, generally yielded similar values for oligosaccharides, sucrose, and galactinol across all matrices. However, higher concentrations of monosaccharides and m-inositol were detected in most cases. The presence of monosaccharides was even more pronounced across all matrices when extractions were performed at 60 °C using 80% ethanol in a sequence of three 45 min extractions, as suggested Gangola et al. [12], a method originally proposed for sugar extraction in chickpea. In some cases, galactose levels were found to be an order of magnitude higher (Table 3). Moreover, this latter method showed significantly lower concentrations of raffinose and galactinol, with losses of up to 75% in verbascose content detected in *P. sativum*. However, when considering the total sugar content (sum of RFOs, sucrose, and monosaccharides), the differences between the methods are smaller and, in some cases, could be attributed to the reproducibility of the analytical procedures, suggesting possible degradation of oligosaccharides. In this regard, long extraction periods at mild temperatures, without ensuring the inactivation of substrate enzymes, could be the cause of the differences observed between the evaluated methods.

On the other hand, the accuracy obtained for the estimation of total sugar content (sum of RFOs, sucrose, and monosaccharides) in two samples from interlaboratory comparisons—one sample of pea (*P. sativum*) and another of soybean (Glycine max)—yielded accuracies of 103% and 101%, respectively, with a Z score < 2, falling within the acceptance ranges of the tests (Table S2).

Precision was assessed through repeatability and reproducibility, using two operators on different days, conducting tests independently (two replicates per operator per day) on the same matrices. Repeatability (r), estimated for each analyst and compound, ranged from 0.2 to 6.8% for dry bean and 1.7–6.7% for pea at concentration levels above 1 mg/g. At these levels, reproducibility (R), evaluated as the relative standard deviation (RSD) between analysts, was ≤8.4% in all cases. For concentrations below 1 mg/g, repeatability and reproducibility values were slightly higher, ranging from 3.5 to 10.6% and 3.4–13.4%, respectively (Table 4).

The Standard Method Performance Requirements for Sugars in Animal Feed, Pet Food, and Human Food, as defined by the AOAC [33], specify in the validation guide that, for the lowest analytical range (0.1–5% *w*/*w*, corresponding to 1–50 mg/g), the method should achieve a maximum repeatability and reproducibility of 7% and 10%, respectively, and an accuracy between 90 and 110%. Based on these criteria, the proposed method meets the validation guide requirements in terms of recovery, repeatability, and reproducibility, and can be considered suitable for characterizing both species.

### 3.2. Soluble Carbohydrates in Local Collections of P. vulgaris and P. sativum

The optimized and validated method was applied to analyze the soluble carbohydrate content in two seed collections of common bean (*P. vulgaris*) and pea (*P. sativum*).

#### 3.2.1. Common Beans

Qualitatively, the soluble carbohydrate profile of the *P. vulgaris* collection consisted of four major sugars (verbascose, stachyose, raffinose, and sucrose), two cyclitols or derivatives (myo-inositol and galactinol), and, occasionally, the presence of galactose and glucose (Table 5). Quantitatively, sucrose and stachyose were the most relevant soluble carbohydrates in this species, each being the predominant sugar in 50% of the samples. Moreover, the average values and concentration ranges in the collection samples were similar for both sugars (25.7 ± 5.1 mg/g for sucrose and 26.0 ± 4.9 mg/g for stachyose), and together they accounted for between 76% and 96% of the total soluble carbohydrates analyzed. Other soluble sugars present in all samples, although at significantly lower concentrations, included raffinose (ranging from 0.8 to 7.4 mg/g) and verbascose (ranging from 0.3 to 1.3 mg/g). In contrast, galactose was only quantified in six samples, and glucose in one of the twelve samples in the panel, while fructose was not detected in any of the analyzed samples. Therefore, the presence of monosaccharides does not appear to be relevant in this species. Thus, although the results show some discrepancies compared to those reported by other authors—such as the exclusion of certain oligosaccharides analyzed in this study or differences in monosaccharide content, which may be attributed to possible degradation during extraction, as previously discussed—the total sugar content in the collection of local *P. vulgaris* varieties, ranging from 47.0 mg/g to 72.1 mg/g, is consistent with the data reported for the species [2,20,21,34,35,36,37,38].

The total content of RFOs (raffinose family oligosaccharides), which are considered by several authors as antinutritional factors due to their association with flatulence and diarrhea, accounted for 47% to 57% of the soluble carbohydrates, with an average concentration of 29.3 mg/g. These values, along with the MD/RFO ratio proposed by Kotha et al. [13], may serve as indicators of the species’ potential to induce flatulence. According to findings reported by these authors, pulses from various species may be grouped into categories based on their flatulence potential, and the common bean samples analyzed in this study could be placed within the groups with medium-to-low flatulence potential. It is important to emphasize that, although such criteria may be useful for evaluating legume quality, ensuring proper sample handling and analytical procedures is essential to preserve RFO integrity. Otherwise, elevated MD/RFO ratios may result from either a reduction in RFOs or an increase in MDs, even when the total sugar content remains unchanged.

#### 3.2.2. Pea

The panel of 34 varieties characterized in this study (21 local varieties and 13 commercial ones) exhibited a qualitative profile similar to that of common beans, consisting primarily of verbascose, stachyose, raffinose, sucrose, galactinol, myo-inositol, galactose, and, occasionally, fructose and glucose (Table 6). However, from a quantitative standpoint, several differences between species should be highlighted. In addition to a higher average total sugar content in the *P. sativum* collection (mean value: 68.8 mg/g), this species showed notably higher concentrations of verbascose, ranging from 8.6 to 38.3 mg/g. Verbascose was the predominant oligosaccharide in 21 out of the 34 samples, while sucrose was predominant in 11 samples, and stachyose in 1. In contrast, stachyose content was lower in *P. sativum*, while sucrose levels were similar in both species, although the variability ranges were higher for both sugars. The remaining soluble carbohydrates were detected at significantly lower concentrations, with average contents below 1 mg/g. The values obtained in this study are of the same order as those reported by other authors for this species [13,29,39,40], although some studies reported raffinose as the predominant sugar in *P. sativum* [25].

From a commercial perspective, sugar content is associated with positive consumer perception and is therefore one of the key traits targeted in breeding programs. In this regard, the collection of commercial *P. sativum* cultivars exhibited significantly higher total sugar content (*p* < 0.01) than local varieties, with mean values of 89.0 mg/g and 56.2 mg/g, respectively. This difference was largely due to a higher sucrose content (*p* < 0.01) in the commercial varieties (mean: 39.3 mg/g) compared to the local ones (mean: 15.2 mg/g), which also resulted in a lower MD/RFO ratio (*p* < 0.01) in the local samples (ratio: 0.38) versus the commercial ones (ratio: 0.79). In any case, it should be noted that some local varieties, such as V1119 (Table 6), which combine favorable MD/RFO ratios and high sugar content, along with good adaptation to local conditions, may serve as a suitable basis for future breeding trials.

#### 3.2.3. Linear Discriminant Analysis

A linear discriminant analysis (LDA) was conducted to identify the soluble carbohydrates that most effectively discriminate among the analyzed collections at both the interspecific (*P. vulgaris* vs. *P. sativum*) and intraspecific (local vs. commercial) levels. For this purpose, based on the dataset comprising the varieties and variables from Table 5 and Table 6, fructose and glucose were initially excluded, as they were considered occasional in both species. Additionally, due to the high correlation of sucrose with the MD and total sugars (r_sucrose/MD_ = 0.98, r_sucrose/total sugars_ = 0.92), the latter were removed from the dataset, as they carried greater associated uncertainty.

**Figure 2:**
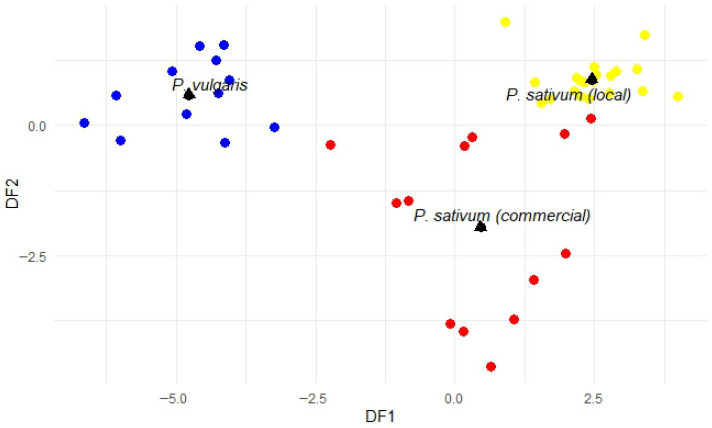
Projection of the pulses (circles) and the centroids of each category (triangles) onto the discriminant function space. *P. vulgaris* (blue), local *P. sativum* (yellow), and commercial *P. sativum* (red).

**Figure 3:**
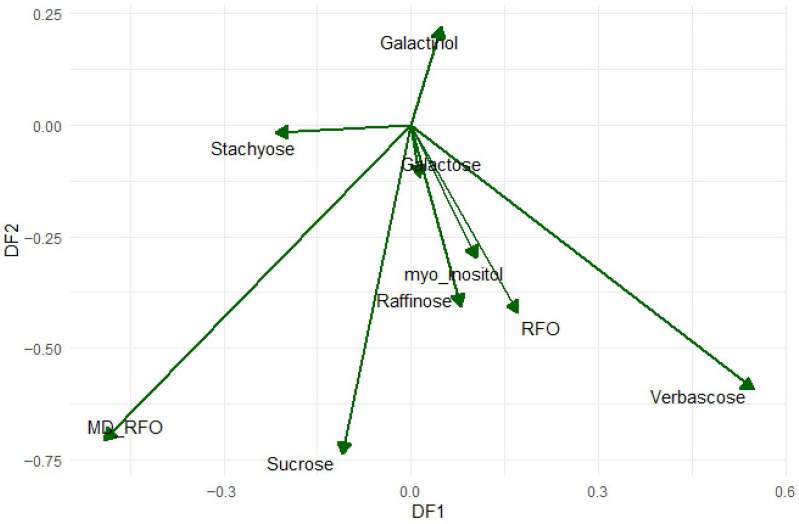
Plot of variable loadings projected onto the discriminant function space.

LDA was performed on a dataset comprising 9 variables and 46 objects, categorized into groups of common beans (*n* = 12), local peas (*n* = 21), and commercial peas (*n* = 13). The model computed two discriminant functions, excluding the variable RFO from the analysis for providing redundant information to the model (Table S3). Figure 2 displays the projection of the discriminant scores for each sample along the two axes and Figure 3 shows the loadings of the variables. Three well-defined groups can be observed: along the *x*-axis, discriminant function 1 (85% of the variance) separates the species, with *P. vulgaris* samples—characterized by higher MD/RFO and lower verbascose—positioned on the left, and *P. sativum* samples—showing lower MD/RFO and higher verbascose—on the right. Meanwhile, discriminant function 2 (15% of the variance) separates local *P. sativum* samples positioned toward the lower end of the *y*-axis, which are associated with higher sucrose levels and MD/RFO ratios compared to commercial *P. sativum* varieties.

When the discriminant functions were used for classification and predictive purposes, 93.5% and 91.3% of the samples were correctly classified and predicted, respectively. Most misclassifications occurred among the commercial *P. sativum* samples, categorized as local (Tables S4 and S5). Despite the limited number of lines analyzed, particularly for *P. vulgaris*, these results highlight the usefulness of soluble carbohydrates as effective tools for the characterization of these pulses and their derived products.

## 4. Conclusions

A method for accurately quantifying soluble carbohydrates in pulses, based on extraction with high-power ultrasound (UAE) and quantification by HPLC–IR, has been successfully developed and validated. The analytical characteristics of the method (repeatability, reproducibility, accuracy, linearity, selectivity, and sensitivity) allow soluble carbohydrates of two diversity panels (*P. vulgaris* and *P. sativum*) to be reliably quantified. Clear differences in soluble carbohydrate profiles were observed between both species, with pea exhibiting higher verbascose levels and greater total sugar content than common bean. Within *P. sativum*, commercial cultivars showed higher sucrose and total soluble carbohydrate contents than local varieties, although several local accessions displayed favorable nutritional profiles with potential application in breeding programs. Soluble carbohydrates have proven to be effective tools for the characterization of pulses, such as peas and common beans, as well as their derived products. These results should be regarded as preliminary, and a comprehensive characterization is required, incorporating greater genetic diversity and cultivation conditions, to establish a robust database for nutritional, technological, and breeding program applications.

## Figures and Tables

**Table 1:** Soluble carbohydrates detected in *Pisum sativum* and *Phaseoulus vulgaris*.

Peak	Compound	t_R_	[M + Na]+	Molecular Formula	Species
1	Ajugose	9.2	1013.3167	C_36_H_62_NaO_31_	2
2	Verbascose *	9.7	851.2639	C_30_H_52_NaO_26_	1, 2
3	Stachyose *	10.3	689.2111	C_24_H_42_NaO_21_	1, 2
4	Raffinose *	11.3	527.1583	C_18_H_32_NaO_16_	1, 2
5	Digalacto-myo-inositol	12.2	527.1583	C_18_H_32_NaO_16_	1, 2
6	Sucrose *	12.8	365.1054	C_12_H_22_NaO_11_	1, 2
7	Galactinol *	14.6	365.1054	C_12_H_22_NaO_11_	1, 2
8	Glucose *	15.8	203.0526	C_6_H_12_NaO_6_	1, 2
9	Galactose *	17.3	203.0526	C_6_H_12_NaO_6_	1, 2
10	Fructose *	18.4	203.0526	C_6_H_12_NaO_6_	1, 2
11	Myo-inositol *	19.1	203.0526	C_6_H_12_NaO_6_	1, 2
12	Mannitol *	21.1	205.0696	C_6_H_14_NaO_6_	1, 2
13	Galactitol *	22.7	205.0701	C_6_H_14_NaO_6_	1, 2
14	Sorbitol *	24.2	205.0699	C_6_H_14_NaO_6_	1, 2

*: verified with pure standard. t_R_: retention time. 1: *P. sativum*; 2: *P. vulgaris*.

**Table 2:** Analytical characteristics of the calibration curves for soluble carbohydrates in pulses.

Compound	Calibration Curve				Instrumental LOD (mg/L)	Instrumental LOQ (mg/L)	Method LOQ (mg/g)
	Linear Range (mg/L)	Slope	Intercept	Correlation Coefficient			
Verbascose (*n* = 7)	15–3000	191.4	−1174	0.9998	6.2	20.7	0.41
Stachyose (*n* = 7)	15–3000	183.8	−151	0.9997	7.8	26.0	0.52
Raffinose (*n* = 7)	15–3000	181.1	−52	0.9999	4.5	15.0	0.30
Sucrose (*n* = 7)	15–3000	201.6	−240	0.9998	6.2	20.7	0.41
Galactinol (*n* = 6)	5–500	192.5	−300	0.9999	2.2	7.5	0.15
Glucose (*n* = 6)	5–500	201.0	−369	0.9999	2.3	7.7	0.15
Galactose (*n* = 6)	5–500	208.3	−320	0.9999	2.0	6.7	0.13
Fructose (*n* = 6)	5–500	212.6	−76	0.9998	2.1	7.0	0.14
Myo-Inositol (*n* = 6)	5–500	239.3	−399	0.9997	4.1	13.7	0.27

*n* = number of points in calibration curve. LOD: limit of detection. LOQ: limit of quantitation.

**Table 3:** Soluble carbohydrate content in pulses under different extraction conditions.

	**V**	**St**	**R**	**S**	**Gmol**	**Glucose**	**Galactose**	**Fructose**	**Myo-** **Inositol**	TSC *
*P. vulgaris*										
m1	1.1 ± 0.0 ab	31.0 ± 2.1 b	2.9 ± 0.1 ab	34.8 ± 2.2	1.6 ± 0.1 b	0.0 ± 0.0 a	0.0 ± 0.0 a	0.0 ± 0.0 a	0.7 ± 0.1 a	69.9
m2	1.2 ± 0.0 b	27.8 ± 2.1 ab	2.4 ± 0.0 a	37.1 ± 1.8	1.6 ± 0.1 b	0.4 ± 0.0 b	0.6 ± 0.1 b	0.5 ± 0.1 b	1.1 ± 0.0 b	70.0
m3	0.9 ± 0.1 a	21.1 ± 4.2 a	4.1 ± 0.9 b	29.7 ± 3.9	0.9 ± 0.1 a	0.1 ± 0.0 a	1.6 ± 0.3 c	0.1 ± 0.0 a	1.1 ± 0.1 b	57.6
*P. sativum*										
m1	21.0 ± 1.3 b	11.2 ± 0.5	3.4 ± 0.2 b	16.8 ± 0.3 a	0.4 ± 0.0 b	0.0 ± 0.0 a	0.4 ± 0.0 a	0.0 ± 0.0 a	0.5 ± 0.0 a	52.8
m2	20.2 ± 0.8 b	11.6 ± 0.4	2.8 ± 0.0 ab	19.7 ± 0.0 b	0.4 ± 0.0 b	0.4 ± 0.0 c	0.8 ± 0.1 a	0.5 ± 0.0 c	0.7 ± 0.0 b	56.0
m3	4.8 ± 0.7 a	11.3 ± 1.4	2.6 ± 0.3 a	19.5 ± 0.9 b	0.2 ± 0.0 a	0.2 ± 0.0 b	10.6 ± 1.1 b	0.2 ± 0.0 b	0.8 ± 0.0 b	49.3
*C. arietinum*										
m1	0.5 ± 0.0 a	21.0 ± 1.6 b	25.1 ± 0.6 b	28.9 ± 2.6 ab	1.7 ± 0.1 b	1.5 ± 0.1 a	0.0 ± 0.0 a	0.0 ± 0.0	0.8 ± 0.1 a	77.1
m2	0.7 ± 0.0 b	19.2 ± 0.7 b	24.6 ± 0.5 b	31.4 ± 0.3 b	1.6 ± 0.0 b	1.8 ± 0.0 b	0.6 ± 0.0 b	0.2 ± 0.0	1.2 ± 0.0 b	78.5
m3	0.7 ± 0.0 b	15.7 ± 0.2 a	21.5 ± 0.3 a	25.4 ± 0.7 a	1.1 ± 0.0 a	1.5 ± 0.0 a	1.2 ± 0.0 c	0.0 ± 0.0	0.9 ± 0.0 a	66.0
*L. culinaris*										
m1	4.1 ± 0.4 b	19.4 ± 1.0 b	15.6 ± 0.8 b	13.9 ± 0.8	1.2 ± 0.0 b	1.0 ± 0.1 a	0.3 ± 0.0 a	0.0 ± 0.0 a	0.4 ± 0.2 a	54.3
m2	4.2 ± 0.4 b	17.1 ± 1.1 b	14.0 ± 0.0 b	13.7 ± 0.0	1.1 ± 0.1 b	2.1 ± 0.0 c	0.6 ± 0.1 a	0.5 ± 0.0 b	0.5 ± 0.0 ab	52.2
m3	1.6 ± 0.1 a	11.4 ± 0.8 a	12.1 ± 0.4 a	14.3 ± 0.4	0.6 ± 0.0 a	1.7 ± 0.1 b	6.9 ± 0.4 b	0.2 ± 0.1 ab	0.7 ± 0.0 b	48.3

V: verbascose, St: stachyose, R: raffinose, S: sucrose, Gnol: galactinol, m1: extraction using high-intensity ultrasound under optimized conditions described in Section 2.3. m2: extraction with water in an ultrasonic bath for 60 min [13]. m3: extraction with 80% ethanol in a water bath at 60 °C, performed in a sequence of three extractions of 45 min each [12]. Different letters for each compound and species indicate statistically significant differences (*p* < 0.05) according to Duncan’s test. TSC: total sugar content. *: total sugar content includes verbascose, stachyose, raffinose, sucrose, glucose, galactose, and fructose.

**Table 4:** Repeatability and reproducibility of the method under optimized conditions in common bean and pea.

	**Verbascose**	**Stachyose**	**Raffinose**	**Sucrose**	**Galactinol**	**Galactose**	**Myo-Inositol**
*Phaseolus vulgaris*							
Operator 1							
Mean value (mg/g, *n* = 2)	1.10	31.00	2.92	34.82	1.63	n.d.	0.68
Repeatability, r (RSD, %)	4.48	6.78	1.94	6.34	6.07		9.43
Operator 2							
Mean value (mg/g, *n* = 2)	1.20	34.88	2.89	37.58	1.73	n.d.	0.73
Repeatability, r (RSD, %)	0.59	0.43	2.94	0.15	3.27		4.81
Reproducibility, R (RSD, %)	6.12	8.35	0.73	5.39	4.21		6.02
*Pisum sativum*							
Operator 1							
Mean value (mg/g, *n* = 2)	21.02	11.21	3.35	16.78	0.40	0.40	0.52
Repeatability, r (RSD, %)	6.19	4.48	5.49	1.73	3.54	10.61	5.79
Operator 2							
Mean value (mg/g, *n* = 2)	23.1	12.05	3.75	16.81	0.42	0.47	0.43
Repeatability, r (RSD, %)	5.42	6.40	5.28	4.96	6.73	6.02	0.56
Reproducibility, R (RSD, %)	6.65	5.11	7.97	0.15	3.45	11.38	13.40

RSD: relative standard deviation. n.d.: not detected.

**Table 5:** Soluble carbohydrate content in the panel of local *P. vulgaris* varieties (expressed in mg/g).

**Variety**	**V**	**St**	**R**	**S**	**Gnol**	**Glucose**	**Galactose**	**Myo-Inositol**	**MD**	**RFO**	**MD/ROF**	Total Sugars *
BGE023180	0.9	21.0	1.4	25.5	0.2	n.d.	0.6	n.d.	26.1	23.3	1.1	49.4
V143	0.9	25.5	2.0	24.6	0.3	n.d.	n.d.	0.5	24.6	28.4	0.9	53.0
V200	0.8	29.4	0.8	23.9	0.8	n.d.	n.d.	0.6	23.9	30.9	0.8	54.9
V201	0.3	24.1	2.0	20.6	0.5	n.d.	n.d.	0.7	20.6	26.5	0.8	47.0
V205	0.8	30.4	0.9	27.0	0.9	n.d.	n.d.	0.8	27.0	32.1	0.8	59.1
V206	1.2	30.9	1.0	24.8	0.4	n.d.	n.d.	0.5	24.8	33.1	0.8	57.9
V207	1.2	24.3	2.5	30.1	0.4	n.d.	n.d.	0.8	30.1	28.1	1.1	58.1
V208	0.6	21.2	5.0	22.1	0.3	n.d.	0.7	0.6	22.8	26.8	0.8	49.6
V209	1.3	20.0	7.4	20.5	n.d.	1.2	2.3	0.5	24.0	28.7	0.8	52.7
V213	0.8	30.8	1.5	38.6	0.4	n.d.	0.5	n.d.	39.1	33.0	1.2	72.1
V288	0.9	29.1	1.1	29.1	0.5	n.d.	0.5	n.d.	29.6	31.0	1.0	60.6
V381	0.4	24.8	4.9	21.4	0.4	n.d.	0.7	0.3	22.0	30.2	0.7	52.2
max	1.3	30.9	7.4	38.6	0.9	1.2	2.3	0.8	39.1	33.1	1.2	72.1
min	0.3	20.0	0.8	20.5	n.d.	n.d.	n.d.	n.d.	20.6	23.3	0.7	47.0
mean	0.8	26.0	2.5	25.7	0.5	n.d.	0.4	0.6	26.2	29.3	0.9	55.6

V: verbascose, St: stachyose, R: raffinose, S: sucrose, Gnol: galactinol, MD: sum of mono- and disaccharides, and RFO: sum of verbascose, stachyose, and raffinose. *: sum of RFO and MD. n.d.: not detected.

**Table 6:** Soluble carbohydrate content in the panel of P. sativum varieties (expressed in mg/g).

**Variety**	**O**	**V**	**St**	R **	S **	Gnol *	**Glucose**	**Galactose**	**F**	**Myo-** **Inositol**	MD **	RFO *	MD/ROF **	Total Sugars ^1,^**
V1062	L	20.4	13.8	n.d.	14.8	0.3	n.d.	0.5	n.d.	0.4	15.3	34.2	0.4	49.5
V1078	L	20.6	12.3	3.5	16.2	0.2	n.d.	0.7	n.d.	0.5	17.0	36.4	0.5	53.4
V1083	L	19.7	19.0	5.6	14.8	0.4	n.d.	0.8	n.d.	0.6	15.6	44.3	0.4	59.9
V1101	L	21.9	17.1	4.3	11.8	0.3	0.2	0.4	n.d.	0.7	12.3	43.3	0.3	55.6
V1105	L	22.7	12.3	2.8	13.1	0.5	0.4	0.4	n.d.	0.8	13.9	37.8	0.4	51.7
V1106B	L	21.7	12.4	3.0	13.6	0.4	n.d.	0.5	n.d.	0.7	14.1	37.1	0.4	51.1
V1107	L	18.4	12.8	2.2	15.4	0.7	n.d.	0.4	n.d.	0.6	15.8	33.4	0.5	49.1
V1108	L	21.6	14.4	3.5	11.8	0.4	n.d.	0.5	n.d.	0.5	12.3	39.5	0.3	51.8
V1109B	L	22.1	11.8	2.0	11.7	0.5	n.d.	0.3	n.d.	n.d.	12.0	35.9	0.3	47.8
V1116	L	25.1	12.9	2.4	13.6	0.5	n.d.	0.4	n.d.	0.5	14.0	40.4	0.3	54.4
V1119A	L	38.3	28.1	7.4	38.7	0.9	n.d.	0.7	n.d.	1.7	39.4	73.8	0.5	113.2
V1122	L	18.5	16.8	4.1	13.8	0.7	n.d.	0.3	n.d.	0.9	14.1	39.4	0.4	53.5
V1123	L	19.1	15.2	3.3	13.9	0.7	n.d.	0.3	n.d.	0.8	14.2	37.5	0.4	51.7
V1127	L	19.3	19.4	5.1	15.2	0.5	n.d.	0.4	n.d.	0.7	15.6	43.9	0.4	59.5
V1129	L	18.4	14.4	4.5	10.8	0.4	0.3	0.4	0.5	0.7	12.1	37.3	0.3	49.4
V1130	L	21.3	12.4	2.3	13.4	0.5	n.d.	0.3	n.d.	0.5	13.8	36.0	0.4	49.7
V1131	L	24.1	13.0	2.5	16.1	0.5	n.d.	0.5	n.d.	0.6	16.5	39.7	0.4	56.2
V1134	L	22.3	13.9	3.2	13.5	0.4	n.d.	0.3	n.d.	0.5	13.8	39.4	0.3	53.2
V1138	L	24.5	14.2	2.8	18.9	0.4	n.d.	0.5	n.d.	0.6	19.4	41.6	0.5	60.9
V1139	L	23.8	16.6	3.4	12.6	0.8	n.d.	0.4	n.d.	0.8	13.0	43.9	0.3	56.9
V1145	L	18.1	15.2	3.8	14.9	0.5	n.d.	0.5	n.d.	0.6	15.4	37.1	0.4	52.5
mean	L	22.0	15.1	3.6	15.2	0.5	n.d.	0.4	n.d.	0.7	15.7	40.6	0.4	56.2
max	L	38.3	28.1	7.4	38.7	0.9	0.4	0.8	0.5	1.7	39.4	73.8	0.5	113.2
min	L	18.1	11.8	n.d.	10.8	0.2	n.d.	0.3	n.d.	n.d.	12.0	33.4	0.3	47.8
S395	C	32.9	19.7	6.2	59.6	0.4	n.d.	0.4	n.d.	0.9	59.9	58.7	1.0	118.7
S396	C	8.6	53.2	10.7	49.3	0.7	n.d.	1.0	n.d.	1.3	50.3	72.5	0.7	122.8
S397	C	35.6	21.8	6.8	50.4	0.4	n.d.	0.8	n.d.	1.2	51.1	64.2	0.8	115.3
S398	C	31.4	20.5	8.0	58.1	0.4	n.d.	0.9	n.d.	1.6	59.1	59.9	1.0	119.0
S399	C	18.6	10.4	3.7	28.2	0.3	n.d.	0.3	n.d.	0.7	28.5	32.7	0.9	61.3
S400	C	14.7	10.5	3.2	15.5	0.3	0.2	0.3	0.2	0.5	16.2	28.4	0.6	44.5
S401	C	23.4	14.0	3.7	17.6	0.4	n.d.	0.4	n.d.	0.5	18.0	41.2	0.4	59.2
S402	C	24.4	10.2	2.4	20.1	0.4	n.d.	0.3	n.d.	0.4	20.4	37.0	0.6	57.4
S403	C	34.2	19.8	6.2	56.5	0.4	n.d.	0.7	n.d.	0.9	57.2	60.2	0.9	117.4
S404	C	19.2	10.0	3.4	28.5	0.4	n.d.	0.4	n.d.	0.6	28.9	32.6	0.9	61.5
S405	C	30.5	18.0	6.0	54.1	0.3	n.d.	0.5	n.d.	0.9	54.6	54.5	1.0	109.1
S406	C	18.2	10.7	2.9	19.7	0.4	n.d.	0.5	n.d.	0.6	20.2	31.8	0.6	52.0
S407	C	36.8	21.2	6.5	53.0	n.d.	n.d.	0.8	n.d.	1.1	53.7	64.5	0.8	118.2
mean	C	25.3	18.5	5.4	39.3	0.4	n.d.	0.6	n.d.	0.9	39.9	49.1	0.8	89.0
max	C	36.8	53.2	10.7	59.6	0.7	0.2	1.0	0.2	1.6	59.9	72.5	1.0	122.8
min	C	8.6	10.0	2.4	15.5	n.d.	n.d.	0.3	n.d.	0.4	16.2	28.4	0.4	44.5
grand mean		23.2	16.4	4.3	24.4	0.5	n.d.	0.5	n.d.	0.8	24.9	43.8	0.5	68.8

O: origin, L: local, and C: commercial. V: verbascose, St: stachyose, R: raffinose, S: sucrose, Gnol: galactinol, F: fructose, MD: sum of mono- and disaccharides, and RFO: sum of verbascose, stachyose, and raffinose. n.d.: not detected. ^1^: sum of RFO and MD. *: significant differences at *p* < 0.05. **: significant differences at *p* < 0.01.

## Data Availability

The original contributions presented in the study are included in the article and Supplementary Materials, further inquiries can be directed to the corresponding author.

## Supplementary Materials

The following supporting information can be downloaded at: https://www.mdpi.com/article/10.3390/foods15020391/s1, Table S1: Soluble carbohydrates at the levels of the factorial design. Mean of two extractions ± standard deviation, expressed in mg/g; Table S2: Method accuracy in samples from interlaboratory assays; Table S3: Standardized coefficients of the canonical discriminant functions; Table S4: Classification matrix by the LDA method; Table S5: Prediction matrix by the LDA method; Figure S1: Soluble carbohydrate concentration (mg/g) and recovery (%) from the first two extractions on *P. vulgaris* seeds; Figure S2: Soluble carbohydrate concentration (mg/g) and recovery (%) from the first two extractions on *P. sativum* seeds.
