# Supplementary material for: Quantitative Assessment of Soluble Carbohydrates in Two Panels of Pulses (Phaseolus vulgaris and Pisum sativum) Using Ultrasound-Assisted Extraction (UAE) and HPLC
Source: Foods. 2026 Jan 21;15(2):391. doi: 10.3390/foods15020391 (PMC12841103; doi:10.3390/foods15020391)
Supplement: Supplementary file 1 [file foods-15-00391-s001.zip › Supplementary Table 1.pdf]

Table S1. Soluble carbohydrates at the levels of the factorial design. Mean of two extractions ± standard deviation, expressed in mg/g .

Time (min)	Mass (g)	Verbascose	Stachyose	Raffinose	Sucrose	Galactinol	Galactose	myo-Inositol
1	0.25	1.2 ± 0.01	34.88 ± 0.15	2.89 ± 0.08	37.58 ± 0.05	1.73 ± 0.06	0.54 ± 0.02	0.73 ± 0.04
2	0.25	1.07 ± 0.05	34.00 ± 1.91	2.85 ± 0.02	36.8 ± 0.60	1.60 ± 0.05	0.48 ± 0.06	0.64 ± 0.05
3	0.25	1.02 ± 0.17	34.85 ± 0.39	2.64 ± 0.33	36.26 ± 1.62	1.59 ± 0.12	0.44 ± 0.03	0.69 ± 0.01
1	0.50	0.92 ± 0.08	32.98 ± 0.19	2.69 ± 0.03	34.98 ± 0.09	1.58 ± 0.01	0.47 ± 0.03	0.62 ± 0.03
2	0.50	0.82 ± 0.07	32.59 ± 0.81	2.71 ± 0.08	35.28 ± 0.86	1.59 ± 0.01	0.46 ± 0.01	0.61 ± 0.04
3	0,50	0,83 ± 0,07	32,19 ± 2,87	2,67 ± 0,15	34,3 ± 3,08	1,57 ± 0,12	0,45 ± 0,03	0,61 ± 0,06
Factor								
Time		n.s.	n.s.	n.s.	n.s.	n.s.	n.s.	n.s.
Mass		**	n.s.	n.s.	n.s.	n.s.	n.s.	*
Time x Mass		n.s.	n.s.	n.s.	n.s.	n.s.	n.s.	n.s.

*: significant differences (p<0.05); **: significant differences (p<0.01); n.s.: not significant
