# Supplementary material for: Quantitative Assessment of Soluble Carbohydrates in Two Panels of Pulses (Phaseolus vulgaris and Pisum sativum) Using Ultrasound-Assisted Extraction (UAE) and HPLC
Source: Foods. 2026 Jan 21;15(2):391. doi: 10.3390/foods15020391 (PMC12841103; doi:10.3390/foods15020391)
Supplement: Supplementary file 1 [file foods-15-00391-s001.zip › Supplementary Table 2.pdf]

Table S2. Method accuracy in samples from interlaboratory assays.

	Verbascose (mg/g)	Stacchyose (mg/g)	Raffinose (mg/g)	Sucrose (mg/g)	Glucose (mg/g)	Galactose (mg/g)	Fructose (mg/g)	Sum (mg/g)	Sum (%)	Reference value, % (*sigma*)	|Z-score|	Accuracy (%)
Pisum sativum												
Mean	11.8	20.2	6.3	13.7	0.6	0.5	0.0	53.1	5.3	5.14 (*1.04*)	0.16	103
RSD (%)	0.1	0.1	0.1	0.1	0.1	0.1	0.1	0.1	0.1			
Glycine max												
Mean	1.5	38.7	10.3	60.5	6.6	0.0	0.3	118.0	11.8	11.288 (*1.165*)	0.44	101
RSD (%)	2.9	2.0	0.6	1.3	0.0	1.0	9.0	1.3	1.3			
