# Supplementary material for: Quantitative Assessment of Soluble Carbohydrates in Two Panels of Pulses (Phaseolus vulgaris and Pisum sativum) Using Ultrasound-Assisted Extraction (UAE) and HPLC
Source: Foods. 2026 Jan 21;15(2):391. doi: 10.3390/foods15020391 (PMC12841103; doi:10.3390/foods15020391)
Supplement: Supplementary file 1 [file foods-15-00391-s001.zip › Supplementary Table 3.pdf]

Table S3. Standardized coefficients of the canonical discriminant functions.

	Discriminant function	
	1	2
Verbascose	0.901	0.474
Stachyose	-0.368	1.193
Raffinose	0.469	-0.786
Sucrose	0.106	-1.842
Galactinol	0.288	0.089
Galactose	-0.136	0.539
myo_inositol	-0.361	0.252
MD_RFO	-0.865	0.365
