# Supplementary material for: Quantitative Assessment of Soluble Carbohydrates in Two Panels of Pulses (Phaseolus vulgaris and Pisum sativum) Using Ultrasound-Assisted Extraction (UAE) and HPLC
Source: Foods. 2026 Jan 21;15(2):391. doi: 10.3390/foods15020391 (PMC12841103; doi:10.3390/foods15020391)
Supplement: Supplementary file 1 [file foods-15-00391-s001.zip › Supplementary Table 4.pdf]

Table S4. Classification matrix by the LDA method.

	Assigned category			
**True category**	**P. vulgaris**	**P. sativum local**	**P. sativum commercial**	**Hits (%)**
**P. vulgaris**	12	0	0	100
**P. sativum local**	0	21	0	100
**P. sativum commercial**	1	2	10	77
**Overall**				93
